# Bibliometric analysis highlights racial diversity of type 2 diabetes research: Global trends from 2010 to 2024

**DOI:** 10.3934/publichealth.2026016

**Published:** 2026-03-05

**Authors:** Jiayue Li, Zeping Liu, Jun Liu

**Affiliations:** 1 School of Mathematics and Statistics, Wuhan University, Wuhan, China; 2 West China School of Medicine, Sichuan University, Chengdu, China; 3 College of Computer Science, Sichuan University, Chengdu, China; 4 Tongji Hospital, Tongji Medical College, Huazhong University of Science and Technology, Wuhan, China

**Keywords:** bibliometric analysis, race, type 2 diabetes, racial disparities, global health

## Abstract

**Background:**

Type 2 diabetes (T2D) affects millions worldwide with marked racial disparities in prevalence, pathophysiology, and outcomes. Despite growing research, the global landscape and focus on racial diversity in T2D research remain unclear.

**Objective:**

To map and characterize research on T2D and race, with a focus on publication. Research design and methods: We performed a bibliometric analysis of 9087 English-language publications on T2D and 5 mutually exclusive racial populations (American Indian, Alaskan Native, or Indigenous; Asian; Black; Native Hawaiian or other Pacific Islander; and White) from 2010 to 2024, retrieved from the Web of Science Core Collection. Using VOSviewer, CiteSpace, and R software, we analyzed publication trends, country and institutional contributions, key authors, keyword clusters, and citation patterns across the racial populations.

**Results:**

Annual publications and citations increased steadily within 15 years. Research output and influence were most frequently associated with White populations, followed by Asian and Black populations. The United States and China contributed the most publications, while international collaboration remained limited but regionally focused. Keyword analysis revealed race specific research emphases, such as gestational diabetes in Indigenous groups and hypertension in Black populations. Studies on Asian and Black populations tended to receive fewer citations and were published in journals with comparatively lower impact factors than those focusing on White populations.

**Conclusions:**

Our study reveals significant diversities in T2D research coverage and impact across racial groups. Enhanced global collaboration and culturally tailored approaches are essential to address racial disparities and improve T2D prevention and care worldwide.

## 1. Introduction

Diabetes mellitus (DM) is one of the most common chronic metabolic disorders. Globally, DM affected 460 million people in 2019, with 80% living in low- and middle-income countries [1]–[3]; by 2024, the number rose to 589 million, and is projected to reach 853 million by 2050 [4]. It significantly shortens life expectancy and increases risks of cardiovascular disease, kidney failure, cancer, amputation, and blindness, particularly among working-age populations [3],[5]. The economic impact is substantial, with global diabetes-related health expenditures exceeding $1.015 trillion in 2024 and projected to reach $1.043 trillion by 2050 [4]. Type 2 diabetes (T2D), which constitutes around 90% of all DM cases, arises from impaired insulin secretion and insulin resistance [6]. Since the 2010s, research has evolved from identifying genetic underpinnings to large-scale, multi-ethnic genomic studies. These studies increasingly acknowledge the importance of racial diversity in disease risk, progression, and treatment outcomes [7].

Race plays a crucial role in T2D disparities. Evidence shows that disease susceptibility, clinical presentation, progression, and response to treatment vary significantly across racially defined groups [8]. For example, distinct metabolic phenotypes and variations in insulin sensitivity and β-cell function have been observed between populations of different racial backgrounds [9]. These diversities are influenced by a combination of genetic, lifestyle and socio-structural factors [10].

Growing research has explored how these factors contribute to racial disparities in T2D. However, the increasing volume and variability of literature necessitate a structured method to synthesize findings and point the future trends of this research field. Bibliometric analysis provides a quantitative approach to evaluate the scientific output, trends, and knowledge structure of a research field by analyzing publications, authors, institutions, keywords, and citations [11]. This method has been widely applied in diverse medical domains, including Chronic obstructive pulmonary disease (COPD) [11], breast cancer [12], exosomes in cardiovascular disease [13], and T cells in atherosclerosis [14]. While researchers conducting bibliometric studies have examined diabetes-related topics, such as immunotherapy in type 1 diabetes and metabolomics [15],[16], there is a notable gap in studies addressing racial differences in T2D research.

To bridge this gap, we conduct a comprehensive bibliometric analysis of global literature from 2010 to 2024, focusing on race in T2D research. We map the publication trends, country and institutional contributions, key authors, thematic focuses, and citation patterns, with a specific focus on comparing the five major racial categories commonly used in public health reporting. By mapping the landscape of racial diversity in T2D studies, we aim to inform researchers and policymakers about trends and gaps, thereby encouraging more precise consideration of race in designing interventions, improving care strategies, and promoting equity in T2D prevention and treatment.

## 2. Materials and methods

### 2.1. Sources of data and approaches to data retrieval

To guarantee the precision and quality of the data, we retrieved information from the Web of Science Core Collection (WOSCC) database, which provides standardized, high-quality metadata for publications. Our major objectives were to uncover the overarching patterns in T2D literature involving racial data and explore the factors associated with racial diversity. This was done by identified using 5 mutually exclusive racial categories [17]: (1) American Indian, Alaskan Native, or Indigenous; (2) Asian; (3) Black; (4) Native Hawaiian or other Pacific Islander; and (5) White. The search terms were TS1 = “race” OR “ethnicity” OR “racial disparities” OR “ethnic groups” OR “American Indian” OR “Alaskan Native” OR “Indigenous” OR “Asian” OR “Black” OR “Native Hawaiian” OR “other Pacific Islander” OR “White”. TS2 = “Non-Insulin-Dependent Diabetes Mellitus” OR “type 2 diabetes”. TS3 = “American Indian” OR “Alaskan Native” OR “Indigenous” or TS3 = “Asian” or TS3 = “Black” or TS3 = “Native Hawaiian” OR “other Pacific Islander” or TS3 = “White”.

**Figure 1.**
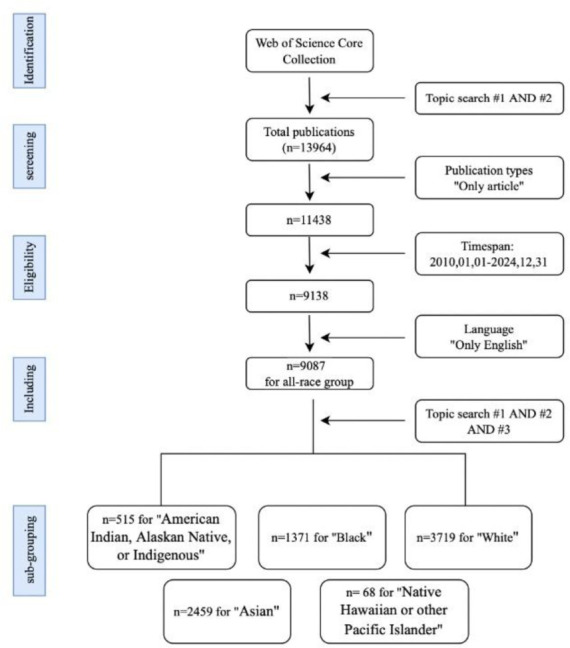
Flowchart of data retrieval and grouping.

As shown in the search flowchart in Figure 1, we first extracted 13,964 publications with the retrieval strategy (TS1 AND TS2). Then, the publication types were set to articles and the time span was set to Jan, 2010–Dec, 2024. The language restriction was English to ensure a consistent and analyzable corpus. Thus, 9087 publications were included in an all-population group. Moreover, to extract publications involving 5 individual populations within the above data, we further supplemented retrieval strategy with “AND TS3”. Each time, TS3 were set as one different population in the above categories. Finally, 515, 2459, 1371, 68, and 3719 publications were included in the American Indian, Alaskan Native, or Indigenous population, Asian population, Black population, Native Hawaiian or other Pacific Islander population, and White population, respectively. The above process was completed by two independent examiners.

### 2.2. Data collection and analysis methods

The full records and references retrieved from WOSCC were exported in plain text format. The Microsoft Excel, VOSviewer (version 1.6.20), CiteSpace (version 6.2.R3), Bibliometrix package in R software (version 4.3.3), and Scimago Graphica (version 1.0.49) were implemented to further refine data and conduct visual assessments of the literature under review. For VOSviewer, the minimum document count required for a node was modified to meet the visualization objectives, with other settings being left at their default values. Specific parameter details of each analysis could be seen in the corresponding results. For CiteSpace, the timeframe ranged from 2010, Jan to 2024, Dec, and the # years per slice was set as 1; for the calculation of relationship strength links, we used a cosine algorithm. To set network pruning functional areas, the Pathfinder algorithm was selected. Moreover, the Log-likelihood rate (LLR) algorithm was used to extract cluster tags. As a bibliometric analysis, we did not measure the methodological quality of individual studies, and metrics such as citation counts and h-indexes reflect research visibility rather than study quality.

We further examined the racial differences in several publication metrics-cited reference counts, WOSCC citation frequency, publication year, journal impact factor (based on the *Journal Citation Reports 2024* published by Clarivate Analytics), and rank of journals based on their impact factor quartile using the White population as the reference category and the other populations as the target population in separate analyses. We used Linear Regression from the *lm* package in R software (version 4.3.3), treating the publication-related trait as the dependent variable and population group as the independent variable (White = 0, other population = 1). Publication year and research area were adjusted for as covariates in the models, where applicable. The false discovery rate (FDR) was used to correct for multiple testing.

## 3. Results

### 3.1. Trends of annual publications and citations

**Figure 2.**
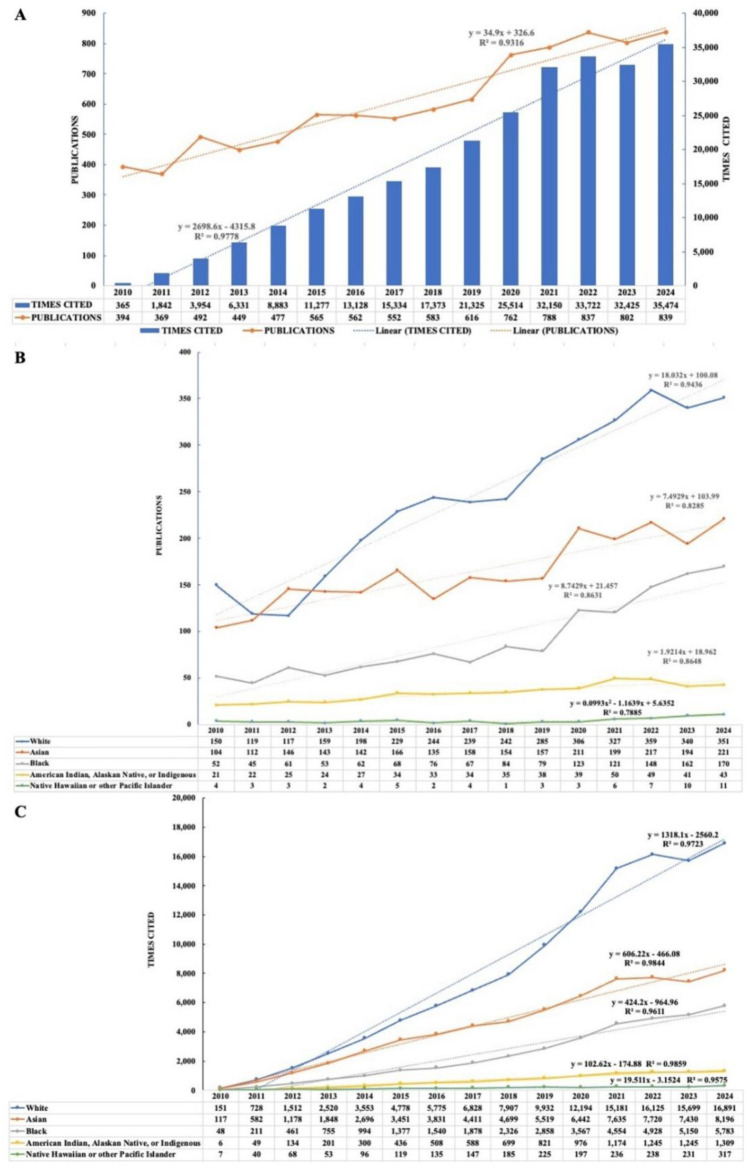
T2D and ethnicity-related publications and citations per year from 2010 to 2024. (A) Number of publications and citations of all populations. (B) Number of publications of five populations. (C) Number of citations of five populations. The fitted curve represents the value over the past 15 years.

A total of 9087 documents focusing on T2D and race were extracted from the WOSCC database, as well as the annual publication and citation data. As depicted in Figure 2A, although there were fluctuations during the period, the overall trend increased. The annual publication count increased by 112.94% between 2010 and 2024, escalating from 394 to 839. A linear equation was used to fit the annual publications increase trend, and the coefficient of determination of the regression curve R2 was 0.9316, indicating that the curve could explain 93.16% of the variation of the dependent variable. The annual times cited showed a more obvious growth trend. In the past five years, the number of citations skyrocketed, accounting for more than 50% of total times cited. Thus, there has been a significant surge in the attention given to the research and reports in this field over the last 15 years. With the fitted curves, we could predict that the number of publications and times cited of articles focusing on T2D and race in 2025 would be about 885 and 38,862, respectively.

The annual publications and citations in five individual populations were also plotted and fitted (Figure 2B and C). From 2010 to 2024, among five populations, the White population had the highest total number of articles at 3665, followed by the Asian population with 2459, and the Native Hawaiian or other Pacific Islander population had the fewest with 68. Similar differences across populations was also found in the number of total citations. In both sets of data, the White population showed the highest increasing trend. Although the Asian population consistently outperformed the Black population across all years from 2010 to 2024, the later had a higher escalating trend of annual publications.

### 3.2. Journal analysis

When analyzing the journal distribution of the literature related to T2D and race, an aggregate of 9087 articles were published in 1694 journals between 2010 and 2024. Supplementary Table 1 shows the top 10 journals according to their H-index in all-population group. Diabetes Care emerged as the most influential in terms of publication, citation, and H-index, G-index, and M-index. Notably, Lancet Diabetes & Endocrinology had only 36 total articles published between 2010 and 2024 but it was cited 6568 times, indicating an obvious extensive citation per article, meaning a significant influence in the field. These 10 journals have been instrumental in facilitating a discourse and dissemination of knowledge within the specialty of T2D and race.

Supplementary Table 2 presents the top 10 most influential journals for T2D research across five populations. For all groups, except American Indian, Alaskan Native, or Indigenous populations, Diabetes Care emerged as the most frequently cited journal. This was followed by Plos One, which published 113 papers for the White population and 103 for the Asian population, although its citation per publication was lower compared to journals such as Diabetologia. Notably, although only five papers were published in Diabetes Care for the Native Hawaiian or Other Pacific Islander group, these achieved a remarkably high average citation rate of 143.8 per publication.

Based on Supplementary Tables 1 and 2, the proportion of publications in Q1 journals among articles published in the top 10 journals across all groups ranged from 38.3% to 68.9%, with most groups having between 40% and 50% of their articles in Q1 journals.

### 3.3. Country/region distribution analysis

**Figure 3.**
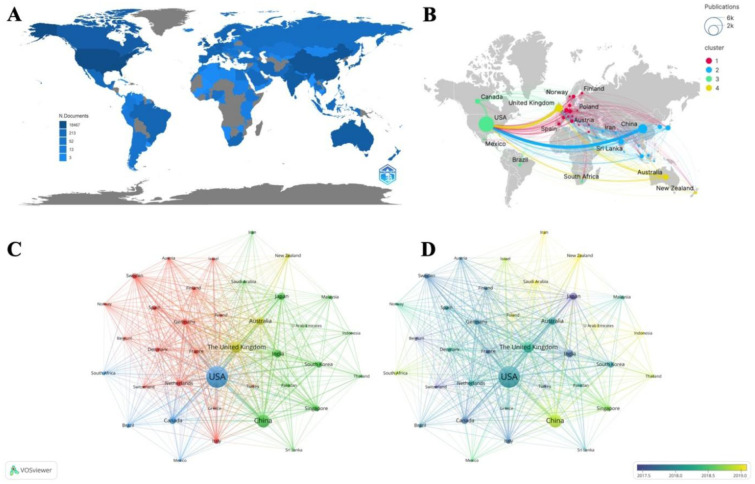
Analysis of national cooperative networks in all populations. (A) Map of country publication made by Bibliometrix. (B) The network visualization of countries that have 5 or more documents (by VOSviewer); the larger the node, the more frequent the publications. (C) The nation overlay visualization with superimposed time documents (by VOSviewer). The color from purple to yellow indicates time from far to near. (D) Map of country collaboration made by Scimago Graphica. Node size indicates the number of publications.

Regarding national contributions to T2D and racial research, significant differences exist in the racial focus among countries. The United States led the field with 4090 publications, 145,218 citations, and an H-index of 180 (Supplementary Table 3 and Figure 3A). Although, based on the publications, the United States and China ranked first and second, respectively, their multiple-country publication percentage (MCP) were relatively low. Oppositely, countries like Germany and Australia completed almost half their publications through international cooperation. The U.S. primarily studied T2D in African American and White populations, with relatively low international collaboration. In contrast, Asian countries such as China, Japan, and South Korea concentrated their T2D research on Asian populations. These countries also exhibited low international collaboration rates. According to the co-authorship network (Figure 3B), the U.S. had the largest node, followed by China, Canada, India, and the UK, highlighting their prolific contributions. The national cooperation clusters (Figure 3C and D) revealed regional partnerships, such as the U.S. with South Africa, and China with India, Iran, Japan, and other Asian countries. This may be related to the geographic and racial proximities.

When examining the top five countries by population (Table 1), the U.S. dominated all categories, particularly excelling in research involving White populations. The UK consistently ranked in the top five across all populations. China focused more on Asian, Black, and White populations. Notably, only 11 countries published papers on T2D in Native Hawaiian or Pacific Islander groups, with the U.S. contributing over 97% of these publications and citations.

Overall, these findings highlight diversities in T2D racial research: Western countries mostly investigate African American and White populations, while East Asian countries focus predominantly on Asian populations.

### 3.4. Author contribution analysis

**Figure 4.**
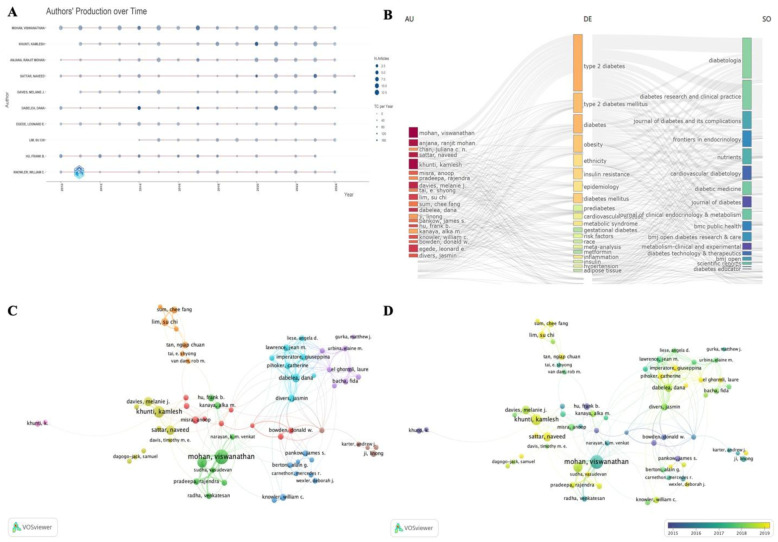
Analysis of the authors' publications and research evolution in the all-population group. (A) Top 10 authors' production over time, which reflects the number of annual publications and citations, analyzed by Bibliometrix. The diameter of the circle corresponds to the publication count (N. Articles), while the color intensity reflects the cumulative citations received. (B) Three field plots made by Bibliometrix. This plot reveals the relationship among authors, keywords, and resources. (C) Author collaboration network made by VOSviewer. The larger the node, the more papers are published. The lines represent collaborations with other authors. The thicker the line, the higher the cooperation frequency. Color represents cluster, and the nodes with the same color belong to the same cluster. (D) Time-based overlay visualization of collaborative relationships among authors (by VOSviewer).

Figure 4 highlights the top 10 most influential authors in T2D and racial research. As shown in Figure 4A and Supplementary Table 4, Mohan Viswanathan from Madras Diabetes Research Foundation and Dr. Mohan's Diabetes Specialties Center, India, was the most productive, with 117 articles and 5127 citations. Hu Frank B. from Harvard University, U.S., stands out with 37 articles and 5090 citations, indicating high citation-per-paper impact. Most of these authors maintained steady publication rates over time. Figure 4B presents a three-field plot linking authors, keywords, and journals. Common research focuses included obesity, insulin resistance, cardiovascular diseases, and metabolic syndrome. Key journals were Diabetologia, Diabetes Research and Clinical Practice, and Journal of Diabetes and Its Complications.

From 46,884 analyzed authors, 82 authors with 17 or more publications were mapped in a collaborative network (Figure 4C), forming nine clusters. Mohan Viswanathan was closely linked to Anjana, Ranjit, Mohan, and Hu Frank B., while Khunti Kamlesh collaborated with Davies Melanie J. and Sattar Naveed. Notably, despite high productivity, Egede Leonard E. showed limited co-authorship. Figure 4D tracks research activity over time, with Mohan Viswanathan emerging around 2017 and Khunti Kamlesh in 2019.

According to Supplementary Table 5, Knowler William C. from George Washington University, U.S. led in American Indian/Alaskan Native research. Mohan Viswanathan from Madras Diabetes Research Foundation and Dr. Mohan's Diabetes Specialties Center, India, dominated Asian-related publications. Egede Leonard E. from the University at Buffalo, U.S., Maskarinec Gertraud from University of Hawaii Cancer Center, U.S., and Khunti Kamlesh from University of Leicester, U.K., were the most active in Black, Native Hawaiian/Pacific Islander, and White population studies, respectively.

**Table 1.** Top 5 productive countries in five populations.

Population	Countries	Publications	Citations	H-index
American Indian, Alaskan Native, or Indigenous	U.S.	203	5008	32
	Australia	147	2414	24
	Canada	66	939	17
	New Zealand	31	528	13
	United Kingdom	21	559	14
Asian	U.S.	677	25,998	74
	China	486	14,575	55
	United Kingdom	398	14,969	62
	India	388	12,453	54
	Japan	233	8088	48
Black	U.S.	987	29,394	76
	United Kingdom	144	5754	36
	China	95	2733	28
	South Korea	71	1924	22
	Canada	47	1611	22
Native Hawaiian or other Pacific Islander	U.S.	66	2360	22
	Germany	8	249	7
	United Kingdom	3	60	3
	Australia	2	32	1
	Japan	2	21	2
White	U.S.	1808	68,899	123
	China	521	12,980	54
	United Kingdom	412	17,027	60
	Japan	190	5820	44
	Canada	167	6879	43

Note: The data is sourced from the Web of Science platform.

### 3.5. Institution distribution analysis

Bibliometrics quantifies research institution performance by analyzing publications and citations. According to the WOSCC database, Supplementary Table 6 lists the top 10 institutions publishing for T2D and race in the all-population group. Harvard University led with the most publications, citations, and highest H-index, followed by the University of California system and the U.S. National Institutes of Health (NIH). Nine of the top 10 institutions were based in the U.S., and one was in the U.K.

Supplementary Table 7 compares the top institutions of five individual populations. For Black and White populations, leading institutions included Harvard, University of California, U.S. Department of Veterans Affairs, Johns Hopkins University, and University of London. For Asian populations, National University of Singapore ranked highest, followed by Madras Diabetes Research Foundation (India) and Harvard University. The University of Hawaii system and Hawaii Cancer Research Center dominated Native Hawaiian or Pacific Islander-related T2D research.

**Figure 5.**
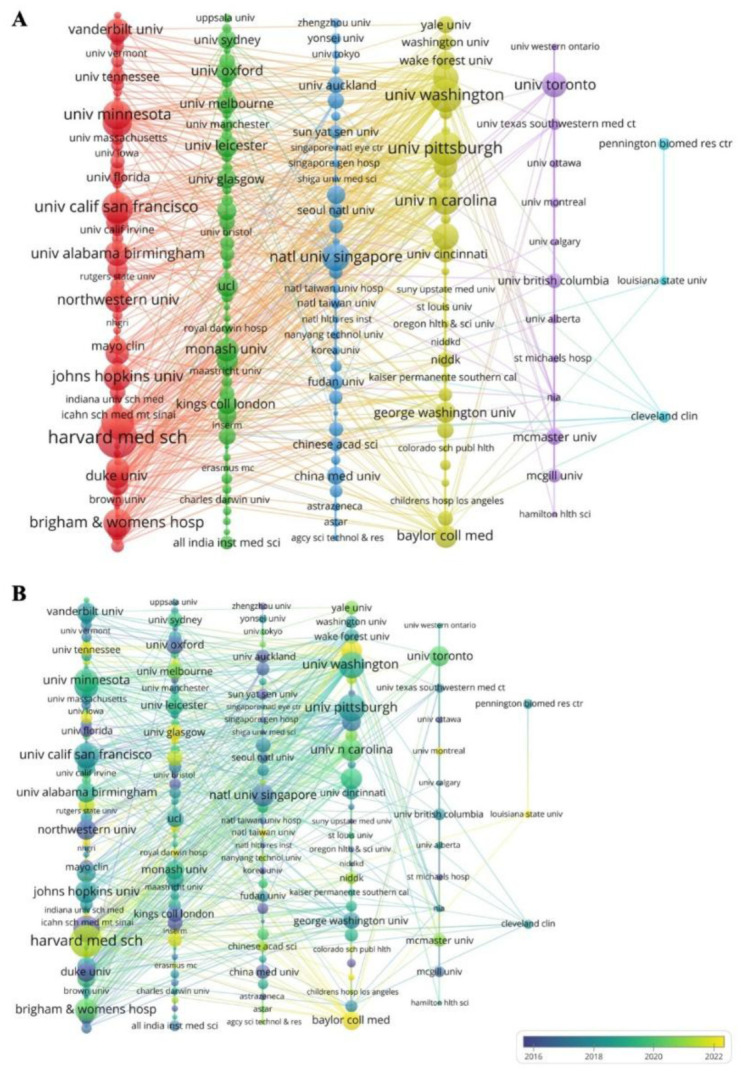
Analysis of an institutional cooperation network of the all-population group. (A) The institution collaboration network is made by VOSviewer. The larger the node, the more papers are published. (B) Time-based overlay visualization of collaborative relationships among institutions by VOSviewer. The color of the circle represents the average time to post. The color from purple to yellow indicates the time from far to near.

Using a 30-publication threshold, 180 institutions are visualized in Figure 5A, categorized into six clusters by color. VOSviewer analyzed subordinate institutions separately, highlighting Harvard Medical School with 243 documents and strong links to U.S. hospitals and universities such as Brigham and Women's Hospital and John Hopkins. Another cluster included Imperial College London, University College London, Monash University, and others. Asian institutions mainly group in a blue cluster with universities like Nanyang Technological University, China Medical University, and Peking University. Figure 5B shows the timeline of institutions' research activities. Purple nodes represent early starters (2016 or earlier) like Harvard University and Duke University; and yellow nodes indicate institutions that began around 2022, such as Harvard Medical School and Baylor College of Medicine.

### 3.6. Keyword analysis

**Figure 6.**
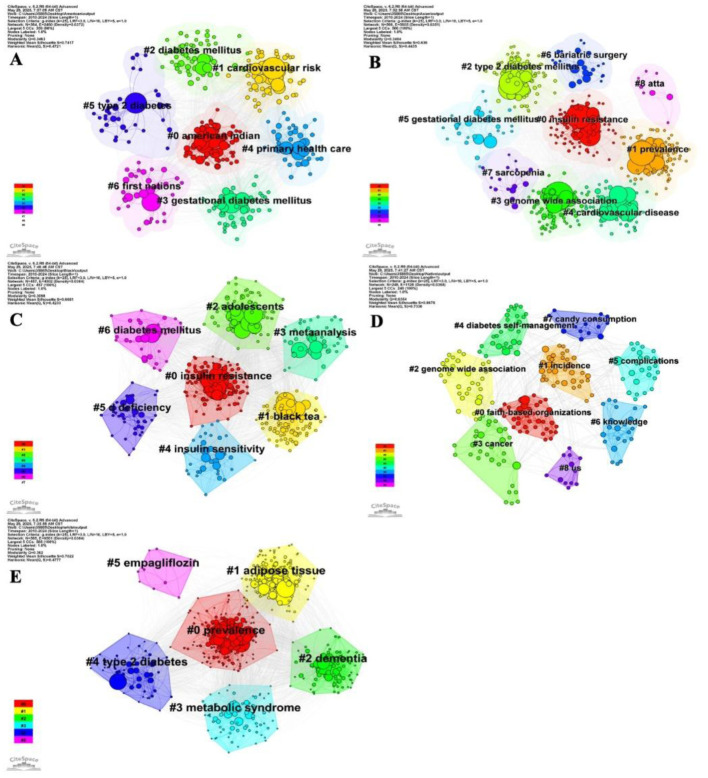
Keyword co-occurrence map created using CiteSpace in five populations. The larger node, the more times the keyword appears. The red circle represents the burst point. (A) American Indian, Alaskan Native, or Indigenous population. (B) Asian population. (C) Black population. (D) Native Hawaiian or other Pacific Islander population. (E) White population.

We first identified the top 10 most frequent keywords in each group (Table 2). Besides the well-known terms related to T2D and race, notable differences in complications were observed across populations: Literature concerning American Indian, Alaskan Native, or Indigenous populations primarily focused on gestational diabetes; studies on Black populations emphasized hypertension; while publications related to White populations frequently mentioned metabolic syndrome, adipose tissue, and inflammation. To explore keyword differences across populations, CiteSpace was applied for cluster analysis within each population (Figure 6). Beyond common keywords like DM, T2D, and race-specific terms, substantial variation was evident: Cardiovascular risk was emphasized among American Indian, Alaskan Native, Indigenous, and Asian groups; prevalence ranked first in Whites and second in Asians; cancer was a key cluster only among Native Hawaiian and other Pacific Islander populations; and dementia was a key cluster unique to Whites.

**Table 2.** Top 10 keywords in the all-population group and five populations.

All populations	American Indian, Alaskan Native, or Indigenous	Asian	Black	Native Hawaiian or other Pacific Islander	White
T2D	T2D	T2D	T2D	T2D	T2D
Diabetes	Diabetes	T2DM	Diabetes	Ethnicity	Obesity
T2DM	Indigenous	Diabetes	DM	T2DM	Diabetes
Obesity	T2DM	DM	Obesity	Obesity	T2DM
DM	Aboriginal	Ethnicity	Ethnicity	Community-based participatory research	Insulin resistance
Insulin resistance	Obesity	Obesity	Race	Pacific islanders	DM
Ethnicity	DM	Meta-analysis	T2DM	Marshallese	Inflammation
Epidemiology	American Indian	Insulin resistance	Epidemiology	Risk factors	Adipose tissue
Metabolic syndrome	Gestational diabetes	Asian Indians	Insulin resistance	Prospective study	Metabolic syndrome
Inflammation	American Indians	Asian	Hypertension	Native Hawaiian	Ethnicity

### 3.7. Statistical comparison between populations for publication variables

Using statistical methods, we compared differences in publication-related variables across populations (Table 3). The results revealed that studies focusing on Asian, American Indian, Alaskan Native, or Indigenous populations had significantly lower WOSCC citation frequency, impact factors, and journal ranks based on impact factor compared to those involving White populations (FDR < 0.05). Moreover, the peak publication year for studies involving Black population was 0.54 years later than those involving the White population (FDR = 3.48 × 10^−4^), while the peak publication year for studies involving the Asian population was 0.31 years earlier than those involving the White population (FDR = 0.019). No significant differences were identified between the White population with the other populations for cited reference counts after FDR correction (FDR ≥ 0.05).

Comparing publication proportions with estimated population-level T2D cases further contextualized the observed diversities (Supplementary Table 8). Within the analyzed literature, studies focusing on Asian populations accounted for 27.06% of publications, while Asians were estimated to represent 63.08% of the global T2D population. In contrast, White populations comprised 40.33% of publications, despite representing only 17.72% of cases.

**Table 3.** Significant differences (FDR < 0.05) between populations for publication results.

Comparison	Publication variables	Beta	SE	P-value	FDR
Asian vs. White	Rank_IF	−0.3	0.03	8.27E–25	1.65E–23
Asian vs. White	Journal_IF	−1.25	0.23	1.08E–07	1.08E–06
Asian vs. White	Times.Cited..WoS.Core	−8.67	1.87	3.79E–06	2.53E–05
Indian vs. White	Rank_IF	−0.26	0.06	5.77E–06	2.89E–05
Black vs. White	Publication.Year	0.54	0.14	8.71E–05	3.48E–4
Indian vs. White	Times.Cited..WoS.Core	−11.84	3.6	0.001	0.003
Asian vs. White	Publication.Year	−0.31	0.11	7.00E–03	0.019
Black vs. White	Cited.Reference.Count	−2.1	0.97	0.03	0.069
Black vs. White	Journal_IF	0.66	0.31	0.031	0.069
Asian vs. White	Cited.Reference.Count	−1.64	0.78	0.036	0.072
Indian vs. White	Journal_IF	−0.93	0.47	0.046	0.083
Indian vs. White	Publication.Year	−0.24	0.22	0.264	0.439
Indian vs. White	Cited.Reference.Count	−1.43	1.57	0.361	0.555
Black vs. White	Times.Cited..WoS.Core	−1.37	2.26	0.544	0.575
Black vs. White	Rank_IF	0.02	0.04	0.486	0.575
Hawaiian vs. White	Times.Cited..WoS.Core	6.09	8.84	0.491	0.575
Hawaiian vs. White	Publication.Year	0.37	0.52	0.476	0.575
Hawaiian vs. White	Journal_IF	0.66	1.1	0.546	0.575
Hawaiian vs. White	Rank_IF	0.1	0.13	0.468	0.575
Hawaiian vs. White	Cited.Reference.Count	0.72	3.91	0.854	0.854

Note: IF: impact factor; Rank IF: rank of journals based on their impact factor quartile, rank = 4 means the top quartile journals; Indian: American Indian, Alaskan Native, or Indigenous; Hawaiian: Native Hawaiian or other Pacific Islander; Times.Cited..WoS.Core: WOSCC citation frequency; FDR: false discovery rate corrected P-value; SE: standard error.

## 4. Discussion

In this bibliometric analysis, we provide a comprehensive overview of 9087 publications from 2010 to 2024 addressing T2D with respect to race, encompassing a broad spectrum of basic, clinical, and epidemiological research. The total citations of these articles reached 259,097, reflecting the substantial scientific interest in the interplay between T2D and racial diversities. This topic appears to be of significant interest, as reflected by the high proportion of publications in top-ranking journals across all groups.

Our findings reveal marked racial diversities in T2D research output and impact. Most of the publications and citations focus on White populations, followed by Asian and Black populations, while Native Hawaiian and other Pacific Islander populations have fewer publications, despite evidence of their disproportionately high diabetes prevalence [18]–[20]. Notably, the number of publications related to Black populations has been rising at a faster rate than those related to Asians, suggesting an increasing recognition of the disease burden within this group [21]. These diversities likely stem from a confluence of factors, including historical inequities in research funding and infrastructure, geographic variation in research capacity, challenges in data collection and participant recruitment within certain communities, and differing priorities within the global research agenda. Consideration of the population-level T2D burden across groups could provide additional context for interpreting differences in research, although precise data for all populations are difficult to obtain.

Geographically, research activity is concentrated in developed regions, particularly North America, Europe, and parts of Asia, with countries like the U.S. and China leading in volume and influence. However, affiliations in regions with high diabetes prevalence, such as parts of South Asia and Africa, tend to have a lower number of publications. Encouragingly, international collaborations exist, but they predominantly occur within adjacent regions, with major contributors like the U.S. and China exhibiting more domestically focused research activities. The lower international collaboration rates observed for the USA and China may be associated with several factors: Large domestic research ecosystems and funding structures that support internal projects; a focus on their own substantial and diverse domestic populations (e.g., African American, Hispanic, and Han Chinese); and, in China's case, linguistic and administrative barriers. In contrast, countries like Germany and Australia, with smaller domestic populations relevant to specific racial research, may actively seek international consortia (e.g., with South Asia or the Pacific Islands) to achieve sufficient sample sizes and expertise, leading to higher MCP ratios. Institutionally, leadership is dominated by centers in the U.S., India, and the UK, with influential researchers such as Dr. Viswanathan Mohan driving substantial contributions in the Asian context. This distribution reflects the research infrastructure and the demographic diversity influencing national research priorities.

A key observation from this bibliometric analysis is the substantial variation in research attention across populations affected by T2D. For instance, populations such as Native Hawaiian and other Pacific Islanders and American Indian and Alaskan Natives, which report high prevalence and early onset of T2D, are represented relatively less in the scientific literature compared to other groups. This discrepancy in research coverage may reflect differences in research prioritization, resource allocation, or scholarly communication patterns. In parallel, the analysis shows differences in academic impact metrics, such as average journal impact factor and citation counts, between studies focusing on Asian and Black populations and those focusing on White populations. These variations may be related to differing research focuses, publication venues, and reach within academic networks.

It should be explicitly stated that this is a bibliometric study, and our findings do not support causal inferences regarding disease mechanisms or clinical outcomes. Discussions of structural social determinants remain at a macro level. Bibliometric data alone cannot establish such causal relationships. Future policy recommendations derived from such analyses should be anchored to standardized indicators; for example, a burden‑attention ratio that compares research output/impact with population size or group‑/region‑specific T2D burden. From a scientometric perspective, these patterns suggest opportunities for more balanced global research coverage. We encourage research funders, international health organizations, and the scientific community to consider potential disparities in attention when formulating research agendas. Efforts to broaden the scope of study populations and to support dissemination in widely accessible publication outlets may contribute to a more comprehensive evidence base for T2D worldwide.

While bibliometric analysis offers valuable macro-level perspectives, certain limitations must be acknowledged. A major limitation is the inherent bias of the WOSCC database toward English-language journals, particularly those from North America and Europe. This means our analysis almost certainly excludes a substantial body of relevant research published in other languages (e.g., Spanish, Portuguese, Chinese, and Japanese) or in regional journals not indexed in WOSCC. Consequently, the research activity and perspectives from many regions, including Latin America, Africa, and parts of Asia, are likely underrepresented in our map of the field. This database bias may limit the global representativeness of our findings when interpreting the geographic and linguistic distribution of research output. Future research could benefit from incorporating additional databases, including regional and language-diverse sources, and exploring socio-economic determinants more granularly. Here, we focus on terminology related to race rather than ethnicity. Categories such as “Hispanic/Latino” or “Chinese” represent ethnic classifications; individuals within these groups may identify with any race. Consequently, our analysis does not extend to interpretations at the ethnicity level. In future research, researchers may further explore ethnic dimensions to broaden the scope of inquiry. Our study maps the evolving landscape of racial and research in T2D, revealing progress and diversity in research focus. By highlighting variations in research attention and impact, it calls for a more inclusive global research agenda that addresses the diverse needs of populations affected by T2D worldwide.

## 5. Conclusions

Our bibliometric analysis reveals significant diversities in the volume, focus, and impact of T2D research across racial groups. To address these gaps and advance health equity, enhanced global collaboration and culturally tailored research approaches are essential. Moreover, the WHO's efforts have encouraged broader international consideration of health equity principles [22]. Moving forward, priority should be given to developing cost-effective, evidence-based interventions while refining frameworks that elucidate how social determinants contribute to racial disparities in T2D. Further progress in this field will benefit from interdisciplinary collaboration and the inclusion of perspectives from non-English literature, which may help reduce systemic bias and broaden the relevance of findings.

## Use of AI tools declaration

During the preparation of this work, the authors used ChatGPT 3.5 to correcting grammatical errors. After using this tool, the authors reviewed and edited the content as needed and take full responsibility for the content of the publication.



## References

[1] Diseases GBD, Injuries C (2020). Global burden of 369 diseases and injuries in 204 countries and territories, 1990–2019: a systematic analysis for the Global Burden of Disease Study 2019. Lancet.

[2] Saeedi P, Petersohn I, Salpea P (2019). Global and regional diabetes prevalence estimates for 2019 and projections for 2030 and 2045: Results from the International Diabetes Federation Diabetes Atlas, 9(th) edition. Diabetes Res Clin Pract.

[3] Chan JCN, Lim LL, Wareham NJ (2021). The Lancet Commission on diabetes: using data to transform diabetes care and patient lives. Lancet.

[4] Federation ID (2025). IDF Diabetes Atlas, 11th Edition: International Diabetes Federation.

[5] Harding JL, Andes LJ, Rolka DB (2020). National and State-Level trends in nontraumatic lower-extremity amputation among U.S. medicare beneficiaries with diabetes, 2000–2017. Diabetes Care.

[6] Galicia-Garcia U, Benito-Vicente A, Jebari S (2020). Pathophysiology of type 2 diabetes mellitus. Int J Mol Sci.

[7] Kreienkamp RJ, Voight BF, Gloyn AL (2023). Genetics of type 2 diabete. Diabetes America.

[8] Pham TM, Carpenter JR, Morris TP (2019). Ethnic differences in the prevalence of type 2 diabetes diagnoses in the UK: Cross-sectional analysis of the health improvement network primary care database. Clin Epidemiol.

[9] Pearson ER (2019). Type 2 diabetes: a multifaceted disease. Diabetologia.

[10] Vasishta S, Ganesh K, Umakanth S (2022). Ethnic disparities attributed to the manifestation in and response to type 2 diabetes: insights from metabolomics. Metabolomics.

[11] Chen M, Zhang Y, Mao Y (2023). Bibliometric analysis of exercise and chronic obstructive pulmonary disease. Int J Chron Obstruct Pulmon Dis.

[12] Yuan J, Liu Y, Zhang T (2024). Traditional Chinese medicine for breast cancer treatment: a bibliometric and visualization analysis. Pharm Biol.

[13] Cui J, Li Y, Zhu M (2023). Analysis of the research hotspot of exosomes in cardiovascular disease: A bibliometric-based literature review. Curr Vasc Pharmacol.

[14] Wei N, Xu Y, Li Y (2022). A bibliometric analysis of T cell and atherosclerosis. Front Immunol.

[15] Jiang Y, Xu Z, Wu Y (2025). Exploring the progress and trends of immunotherapy for type 1 diabetes: A comprehensive bibliometric analysis spanning nearly two decades. Obes Rev.

[16] Li H, Li L, Huang QQ (2024). Global status and trends of metabolomics in diabetes: A literature visualization knowledge graph study. World J Diabetes.

[17] Wei S, Le N, Zhu JW (2022). Factors associated with racial and ethnic diversity among heart failure trial participants: A systematic bibliometric review. Circ Heart Fail.

[18] Steinbrecher A, Erber E, Grandinetti A (2012). Physical activity and risk of type 2 diabetes among Native Hawaiians, Japanese Americans, and Caucasians: the Multiethnic Cohort. J Phys Act Health.

[19] Uchima O, Wu YY, Browne C (2019). Disparities in diabetes prevalence among Native Hawaiians/other Pacific Islanders and Asians in Hawai'i. Prev Chronic Dis.

[20] Kirtland KA, Cho P, Geiss LS (2015). Diabetes among Asians and Native Hawaiians or other Pacific Islanders--United States, 2011–2014. MMWR Morb Mortal Wkly Rep.

[21] Lin J, Thompson TJ, Cheng YJ (2018). Projection of the future diabetes burden in the United States through 2060. Popul Health Metr.

[22] Yao Q, Li X, Luo F (2019). The historical roots and seminal research on health equity: a referenced publication year spectroscopy (RPYS) analysis. Int J Equity Health.

