# Supplementary material for: Bibliometric analysis highlights racial diversity of type 2 diabetes research: Global trends from 2010 to 2024
Source: AIMS Public Health. 2026 Mar 5;13(1):289–305. doi: 10.3934/publichealth.2026016 (PMC13084391; doi:10.3934/publichealth.2026016)
Supplement: Supplementary file 1 [file publichealth-13-01-016-s001.pdf]

Public Health

AIMS Public Health, 13(1): 289–305.
DOI: 10.3934/publichealth.2026016
Received: 16 September 2025
Revised: 28 December 2025
Accepted: 13 January 2026
Published: 05 March 2026

http://www.aimspress.com/journal/aimsph

Research article

Bibliometric analysis highlights racial diversity of type 2 diabetes research: Global trends from 2010 to 2024

Jiayue Li[1,†], Zeping Liu[2,3,†] and Jun Liu[4,*]

[1] School of Mathematics and Statistics, Wuhan University, Wuhan, China
[2] West China School of Medicine, Sichuan University, Chengdu, China
[3] College of Computer Science, Sichuan University, Chengdu, China
[4] Tongji Hospital, Tongji Medical College, Huazhong University of Science and Technology, Wuhan, China

* **Correspondence:** Email: drjunliu@hust.edu.cn.

† These two authors contributed equally.

Supplementary

Table 1. Top 10 most influential journals in all populations.

Journals	Impact factor in 2024	H-index	G-index	M-index	Quartile rankings	Citation	Publication	Citation Per Publication
Diabetes Care	14.8	74	120	4.625	1	18,297	321	41.01
Diabetologia	8.4	53	74	3.313	1	7422	181	31.58
Plos One	2.9	50	78	3.125	3	9285	294	67.91
Diabetes	6.2	49	82	3.063	1	6995	103	40.09
Journal of Clinical Endocrinology & Metabolism	5	36	68	2.25	2	5011	125	21.48
Diabetes Research and Clinical Practice	6.1	30	50	1.875	1	3502	163	19.51
Diabetic Medicine	3.2	30	43	1.875	3	2731	140	182.44
Lancet Diabetes & Endocrinology	44	30	36	2.308	1	6568	36	22.75
Scientific Reports	3.8	29	48	2.231	2	2662	117	23.86
BMJ Open	2.4	27	45	1.8	4	2147	90	41.01

Table 2. Top 10 most influential journals in five individual populations.

Populations	Journals	Journal Impact factor 2024	H-index	G-index	M-index	Quartile rankings	Citation	Publication	Citation Per Publication
American Indian, Alaskan Native, or Indigenous	*DIABETES RESEARCH AND CLINICAL PRACTICE*	6.1	10	17	0.625	1	518	17	30.47
	DIABETOLOGIA	8.4	8	10	0.5	1	286	10	28.60
	PLOS ONE	2.9	8	13	0.533	3	481	13	37.00
	BMC HEALTH SERVICES RESEARCH	2.7	7	9	0.5	3	170	9	18.89
	BMC PUBLIC HEALTH	3.5	7	11	0.438	3	150	11	13.64
	CANADIAN JOURNAL OF DIABETES	2.3	7	14	0.583	4	196	15	13.07
	DIABETES EDUCATOR	-	7	8	0.583	-	133	8	16.63
	MEDICAL JOURNAL OF AUSTRALIA	6.7	7	8	0.438	1	198	8	24.75
	BMJ OPEN	2.4	6	9	0.4	4	140	9	15.56
	DIABETES CARE	14.8	6	10	0.375	1	181	10	18.10
Asian	*DIABETES CARE*	14.8	36	68	2.25	1	5483	68	80.63
	PLOS ONE	2.9	33	47	2.063	3	2841	103	27.58
	DIABETOLOGIA	8.4	28	42	1.75	1	1891	56	33.77
	DIABETES RESEARCH AND CLINICAL PRACTICE	6.1	22	32	1.375	1	1304	72	18.11
	DIABETIC MEDICINE	3.2	21	35	1.313	3	1333	55	24.24
	DIABETES OBESITY & METABOLISM	5.4	17	27	1.133	2	1060	27	39.26
	DIABETES TECHNOLOGY & THERAPEUTICS	5.7	16	23	1	2	606	33	18.36
	JOURNAL OF DIABETES	3	16	24	1.067	3	645	34	18.97
	JOURNAL OF DIABETES INVESTIGATION	3.1	16	29	1.143	3	896	35	25.60
	JOURNAL OF DIABETES AND ITS COMPLICATIONS	2.9	15	22	1	3	554	29	19.10
Black	*DIABETES CARE*	14.8	33	54	2.063	1	3090	75	41.20
	DIABETIC MEDICINE	3.2	13	20	0.813	3	430	21	20.48
	DIABETOLOGIA	8.4	13	23	0.929	1	563	23	24.48
	JAMA NETWORK OPEN	10.5	13	27	1.857	1	1066	27	39.48

	AMERICAN JOURNAL OF CLINICAL NUTRITION	6.5	12	15	0.75	1	1197	15	79.80
	DIABETES RESEARCH AND CLINICAL PRACTICE	6.1	11	17	0.786	1	306	21	14.57
	JOURNAL OF CLINICAL ENDOCRINOLOGY & METABOLISM	5	11	19	0.688	2	977	19	51.42
	JOURNAL OF DIABETES AND ITS COMPLICATIONS	2.9	11	18	0.688	3	331	20	16.55
	JOURNAL OF GENERAL INTERNAL MEDICINE	4.3	10	18	0.625	2	324	18	18.00
	METABOLISM-CLINICAL AND EXPERIMENTAL	10.8	10	12	0.667	1	415	12	34.58
Native Hawaiian or other Pacific Islander	*DIABETES CARE*	14.8	4	5	0.25	1	719	5	143.80
	JAMA NETWORK OPEN	10.5	4	6	0.8	1	89	6	14.83
	ASIA-PACIFIC JOURNAL OF PUBLIC HEALTH	1.4	2	2	0.182	4	27	2	13.50
	BMC PUBLIC HEALTH	3.3	2	2	0.333	3	17	2	8.50
	DIABETOLOGIA	8.4	2	2	0.182	1	108	2	54.00
	EUROPEAN JOURNAL OF CLINICAL NUTRITION	3.6	2	2	0.2	3	24	2	12.00
	JOURNAL OF NUTRITION	3.7	2	2	0.125	2	117	2	58.50
	PLOS GENETICS	4	2	2	0.125	2	168	2	84.00
	PUBLIC HEALTH NUTRITION	3.0	2	2	0.133	3	88	2	44.00
	AMERICAN JOURNAL OF HEALTH BEHAVIOR	2.0	1	1	0.111	4	12	1	12.00
White	*DIABETES CARE*	14.8	46	81	2.875	1	7059	127	55.58
	DIABETOLOGIA	8.4	41	60	2.563	1	4039	95	42.52
	PLOS ONE	2.9	38	59	2.375	3	3924	113	34.73
	DIABETES	6.2	33	64	2.063	1	4326	64	67.59
	OBESITY	4.2	21	36	1.313	2	1715	36	47.64
	SCIENTIFIC REPORTS	3.8	21	37	1.615	2	1445	48	30.10
	DIABETIC MEDICINE	3.2	19	33	1.188	3	1256	56	22.43
	JOURNAL OF CLINICAL ENDOCRINOLOGY & METABOLISM	5	19	32	1.188	2	1106	46	24.04
	AMERICAN JOURNAL OF PHYSIOLOGY-ENDOCRINOLOGY AND METABOLISM	4.2	18	23	1.125	2	1425	23	61.96
	JAMA NETWORK OPEN	10.5	18	36	2.25	1	1366	39	35.03

Note: Data is from Bibliometrix and listed according to the H-index.

Table 3. Top 10 productive countries in all populations.

Country	Articles	Citation	H-index	MCP %
USA	4090	145216	180	18.7
CHINA	1271	31715	79	22.6
UNITED KINGDOM	956	43409	93	35.7
AUSTRALIA	546	20038	67	43.8
INDIA	540	15603	60	25.1
CANADA	461	15361	61	34.0
JAPAN	459	15048	62	18.7
SOUTH KOREA	328	10697	54	18.8
GERMANY	299	13449	62	54.6
NETHERLANDS	299	13298	56	42.3

Note: MCP: multiple-country publication (data from R software). Data of article and citation value comes from VOSviewer. Data of H index comes from Web of Science platform.

Table 4. Top 10 most influential authors of the publications in all populations.

Authors	Country/Institution	Publications	Citations	H-index	G-index	M-index	Start
MOHAN VISWANATHAN	India/ Dr. Mohan's Diabetes Specialities Centre	117	5127	33	69	2.063	2010
KHUNTI KAMLESH	UK/University of Leicester	72	2440	26	48	1.733	2011
ANJANA RANJIT MOHAN	India/ Dr. Mohan's Diabetes Specialities Centre	64	1940	23	43	1.438	2010
SATTAR NAVEED	UK/University of Glasgow	51	2752	27	51	1.688	2010
DAVIES MELANIE J.	UK/University of Leicester	47	944	19	29	1.267	2011
DABELEA DANA	USA/ University of Colorado Anschutz Medical Campus	43	5244	22	43	1.375	2010
EGEDE LEONARD E.	USA/Medical College of Wisconsin	42	797	17	27	1.063	2010
LIM SU CHI	Singapore/National University of Singapore	39	775	16	26	1.333	2014
HU FRANK B.	USA/Harvard University	37	5090	29	37	1.813	2010
KNOWLER WILLIAM C.	USA/National Institute of Diabetes and Digestive and Kidney Diseases	36	1150	18	33	1.125	2010

Notes: H-index, G-index and M-index are metrics used to quantify the impact of a researcher. Start means the earliest publications year in the last 15 years.

Table 5. Top 10 most influential authors of the publications in five individual populations.

Populations	Author	Publications	Citations	H-index	G-index	M-index	Start
American Indian, Alaskan Native, or Indigenous	O'DEA KERIN	15	251	12	15	0.75	2010
	KNOWLER WILLIAM C.	20	308	11	17	0.688	2010
	WALLS MELISSA L.	16	330	11	16	0.917	2014
	MAPLE-BROWN LOUISE J.	17	226	10	15	0.625	2010
	HANSON ROBERT L.	18	261	9	16	0.563	2010
	NELSON ROBERT G.	11	245	9	11	0.563	2010
	ARONSON BENJAMIN D.	10	159	8	10	0.667	2014
	CONNORS CHRISTINE	14	157	8	12	0.615	2013
	SHAW JONATHAN E.	16	173	8	12	0.889	2017
	BARZI FEDERICA	10	116	7	10	1	2019
Asian	MOHAN VISWANATHAN	106	4607	31	66	1.938	2010
	ANJANA RANJIT MOHAN	59	1661	21	40	1.313	2010
	KHUNTI KAMLESH	42	1176	19	34	1.267	2011
	SATTAR NAVEED	33	1454	19	33	1.188	2010
	MISRA ANOOP	35	1017	18	31	1.2	2011
	TAI E. SHYONG	29	2899	17	29	1.063	2010
	JI LINONG	26	1661	16	26	1.067	2011
	RADHA VENKATESAN	27	1816	16	27	1	2010
	SIM XUELING	25	3023	16	25	1.067	2011
	VAN DAM ROB M.	21	2115	16	21	1.067	2011
Black	PALMER JULIE R.	22	1046	16	22	1	2010
	EGEDE LEONARD E.	28	519	15	22	0.938	2010
	ROSENBERG LYNN	20	1020	15	20	0.938	2010
	SELVIN ELIZABETH	14	746	12	14	0.8	2011
	DIVERS JASMIN	11	3017	11	11	0.733	2011
	GOFF LOUISE M.	16	209	11	14	1.375	2018
	DABELEA DANA	10	2974	10	10	0.833	2014
	GEBREGZIABHER MULUGETA	13	295	10	13	0.625	2010
	GOEDECKE JULIA H.	14	490	10	14	0.667	2011
	LAWRENCE JEAN M.	10	2974	10	10	0.833	2014
Native Hawaiian or other Pacific Islander	MASKARINEC GERTRAUD	15	598	11	15	0.688	2010
	KOLONEL LAURENCE N.	10	595	10	10	0.625	2010
	LE MARCHAND LOIC	13	662	10	13	0.625	2010
	HAIMAN CHRISTOPHER A.	10	564	9	10	0.563	2010
	GRANDINETTI ANDREW	7	252	6	7	0.375	2010
	WILKENS LYNNE R.	8	527	6	8	0.375	2010
	JACOBS SIMONE	5	211	5	5	0.455	2015
	MORIMOTO YUKIKO	5	192	5	5	0.333	2011
	HSU WILLIAM C.	4	405	4	4	0.308	2013
	STEINBRECHER ASTRID	4	105	4	4	0.267	2011

Continued on next page

Populations	Author	Publications	Citations	H-index	G-index	M-index	Start
White	KHUNTI KAMLESH	34	1385	18	34	1.2	2011
	SATTAR NAVEED	30	1869	18	30	1.125	2010
	BIESSELS GEERT JAN	16	1520	16	16	1	2010
	HU FRANK B.	18	2323	16	18	1	2010
	DAVIES MELANIE J.	26	576	15	23	1	2011
	ARNER PETER	17	607	13	17	1.083	2014
	BOWDEN DONALD W.	16	424	13	16	1	2013
	DABELEA DANA	20	3071	13	20	0.813	2010
	GERSTEIN HERTZEL C.	14	997	13	14	0.867	2011
	LAWRENCE JEAN M.	16	2965	13	16	0.813	2010

Note: Data is from Bibliometrix and listed according to the H-index.

Table 6. Top 10 productive institutions in all populations.

Institute	Publications	Times cited	Average citations	H-index
Harvard University	584	34,134	58.45	91
University of California system	497	25,921	52.15	74
National Institution of Health USA	289	22,341	77.3	65
US Department of Veterans Affairs	286	10,957	38.31	53
University of Texas System	284	12,423	43.74	48
University of London	274	17,706	64.62	57
Pennsylvania commonwealth system of higher education	259	14,533	56.11	58
University system of Ohio	247	9822	39.77	51
Wake Forest University	228	15,843	69.49	58
Johns Hopkins University	223	13,235	59.35	54

Note: Data from WOSCC.

Table 7. Top 10 productive institutions in five individual populations.

American Indian, Alaskan Native, or Indigenous	Asian	Black	Native Hawaiian or other Pacific Islander	White
Charles Darwin University	National University of Singapore	Harvard University	University of Hawaii system	Harvard University
Menzies School of Health Research	Madras Diabetes Research Foundation	US Department of Veterans Affairs	Cancer research center of Hawaii	University of California system
National Institutes of Health USA	Harvard University	University of California system	University of California system	US Department of Veterans Affairs
University of Melbourne	University of California system	Pennsylvania commonwealth system of higher education	University of South California	University of Texas System
Monash University	University of London	John Hopkins University	Harvard University	University of London
University of South Australia	University of Leicester	University of London	University of Arkansas system	University system of Ohio
University of Colorado system	Imperial College London	University of Pittsburgh	Kaiser Permanente	Pennsylvania commonwealth system of higher education
University of Sydney	Agency for Science Technology Research	University system of Ohio	National Institutes of Health USA	National Institutes of Health USA
University of Queensland	University of Oxford	National Institutes of Health USA	University of Washington	Wake Forest University
University of California System	Chinese University of Hongkong	University of Alabama system	Emory University	Johns Hopkins University

Table 8. Research output and estimated global T2D burden by populations.

Populations	Publication Share	Estimated global population proportion	T2D Prevalence	Estimated proportion of global T2D population*
American Indian, Alaskan Native, or Indigenous	5.67%	<5%	16% [1]	7.14%
Asian	27.06%	58%–60%	10.47% [2]	63.08%
Black	14.49%	14%	12.5% [1]	17.87%
Native Hawaiian or other Pacific Islander	0.75%	0.1%–0.2%	11.7% [3]	0.18%
White	40.33%	11%–16%	8.5% [1]	17.72%

Note: *Estimated global T2D population proportion for each race was calculated as each race's global population proportion (using the midpoint for any range values, e.g., 58%–60%) multiplied by its T2D prevalence, divided by the sum of all five races' population proportion multiplied by their respective T2D prevalence.

References

1. The GlobalEconomy.com (2024) Diabetes prevalence in Asia (% of adults ages 20–79). [cited 2025 December 14]. Available from: https://www.theglobaleconomy.com/rankings/diabetes_prevalence/Asia/.

2. Centers for Disease Control and Prevention (2025) National Diabetes Statistics Report. [cited 2025 December 14]. Available from: https://www.cdc.gov/diabetes/php/data-research/.
3. Centers for Disease Control and Prevention (2023) National Diabetes Statistics Report, 2020–2023: Estimates of diabetes and its burden in the United States. [cited 2025 December 14]. Available from: https://www.cdc.gov/diabetes/php/data-research/index.

AIMS Press

© 2026 the Author(s), licensee AIMS Press. This is an open access article distributed under the terms of the Creative Commons Attribution License (http://creativecommons.org/licenses/by/4.0)
